# Cesarean Section, Formula Feeding, and Infant Antibiotic Exposure: Separate and Combined Impacts on Gut Microbial Changes in Later Infancy

**DOI:** 10.3389/fped.2017.00200

**Published:** 2017-09-26

**Authors:** Farzana Yasmin, Hein Min Tun, Theodore Brian Konya, David S. Guttman, Radha S. Chari, Catherine J. Field, Allan B. Becker, Piush J. Mandhane, Stuart E. Turvey, Padmaja Subbarao, Malcolm R. Sears, CHILD Study Investigators, S. S. Anand, James A. Scott, Irina Dinu, Anita L. Kozyrskyj

**Affiliations:** ^1^Department of Public Health Sciences, University of Alberta, Edmonton, AB, Canada; ^2^Department of Pediatrics, University of Alberta, Edmonton, AB, Canada; ^3^Dalla Lana School of Public Health, University of Toronto, Toronto, ON, Canada; ^4^Centre for the Analysis of Genome Evolution and Function, University of Toronto, Toronto, ON, Canada; ^5^Department of Obstetrics and Gynecology, University of Alberta, Edmonton, AB, Canada; ^6^Department of Agricultural, Food and Nutritional Sciences, University of Alberta, Edmonton, AB, Canada; ^7^Department of Pediatrics and Child Health, University of Manitoba, Winnipeg, MB, Canada; ^8^Department of Pediatrics, University of British Columbia, Vancouver, BC, Canada; ^9^Department of Pediatrics, University of Toronto, Toronto, ON, Canada; ^10^Department of Medicine, McMaster University, Hamilton, ON, Canada

**Keywords:** infant gut microbiota, significance analysis of microarrays, cesarean birth, breastfeeding, antibiotic use, food sensitization

## Abstract

Established during infancy, our complex gut microbial community is shaped by medical interventions and societal preferences, such as cesarean section, formula feeding, and antibiotic use. We undertook this study to apply the significance analysis of microarrays (SAM) method to quantify changes in gut microbial composition during later infancy following the most common birth and postnatal exposures affecting infant gut microbial composition. Gut microbiota of 166 full-term infants in the Canadian Healthy Infant Longitudinal Development birth cohort were profiled using 16S high-throughput gene sequencing. Infants were placed into groups according to mutually exclusive combinations of birth mode (vaginal/cesarean birth), breastfeeding status (yes/no), and antibiotic use (yes/no) by 3 months of age. Based on repeated permutations of data and adjustment for the false discovery rate, the SAM statistic identified statistically significant changes in gut microbial abundance between 3 months and 1 year of age within each infant group. We observed well-known patterns of microbial phyla succession in later infancy (declining Proteobacteria; increasing Firmicutes and Bacteroidetes) following vaginal birth, breastfeeding, and no antibiotic exposure. Genus *Lactobacillus, Roseburia*, and *Faecalibacterium* species appeared in the top 10 increases to microbial abundance in these infants. Deviations from this pattern were evident among infants with other perinatal co-exposures; notably, the largest number of microbial species with unchanged abundance was seen in gut microbiota following early cessation of breastfeeding in infants. With and without antibiotic exposure, the absence of a breast milk diet by 3 months of age following vaginal birth yielded a higher proportion of unchanged abundance of *Bacteroidaceae* and *Enterobacteriaceae* in later infancy, and a higher ratio of unchanged *Enterobacteriaceae* to *Alcaligenaceae* microbiota. Gut microbiota of infants born vaginally and exclusively formula fed became less enriched with family *Veillonellaceae* and *Clostridiaceae*, showed unchanging levels of *Ruminococcaceae*, and exhibited a greater decline in the *Rikenellaceae/Bacteroidaceae* ratio compared to their breastfed, vaginally delivered counterparts. These changes were also evident in cesarean-delivered infants to a lesser extent. The clinical relevance of these trajectories of microbial change is that they culminate in taxon-specific abundances in the gut microbiota of later infancy, which we and others have observed to be associated with food sensitization.

## Introduction

The human gut microbiome is assembled throughout infancy with an age-specific succession of microbes after birth. This assembly is characterized by increasing colonization with bifidobacteria and microbes belonging to the Firmicutes and Bacteroidetes phyla, and depletion of the first colonizers, Enterobacteria (1–7). With the introduction of solids, enrichment is also seen with the beta-Proteobacteria. Cesarean delivery and maternal intrapartum antibiotic prophylaxis delay this trajectory of microbial deposition, while formula feeding can modify it (1, 3). As evident with outcomes of expected weight gain after birth (8), early presence of Enterobacteria in the gut is required for functions, such as infant growth. Yet, continued colonization with members of the Enterobacteria family versus lower enrichment with Bacteroidetes has been associated with greater risk for adiposity in toddlers (5) and food sensitization at age of 1 year (9, 10). These observations point to the importance of trajectories of microbial taxon increases and decreases for normal infant development and freedom from disease.

Our understanding of gut microbial development comes mainly from descriptive studies of taxon abundance with increasing infant age in the presence and absence of common perinatal exposures. Next generation sequencing platforms enable a more complete detection of microbes in infant stool samples (11) and have considerably advanced our knowledge on which microbial compositional patterns are associated with an increased risk of metabolic and immune-related disease (12). Yet many questions remain unanswered. How much of a deviation from the gut microbial succession of a vaginally born, antibiotic-free and breastfed infant is sufficient to predict an outcome such as food sensitization? What attribute of the deviation, for example, delayed colonization with *Bacteroides* species (or fewer increases to abundance) or continued colonization with Enterobacteria (or fewer reductions in abundance), is most relevant to aberrant infant development? Does the age-specific abundance of a gut microbe or the magnitude of change in microbial abundance equally predict food sensitization? Does the number of involved species constitute a risk factor in a deviation from the norm? The pursuit of answers to these important questions requires a meaningful method of quantifying trajectories of microbial taxon increases and decreases during infancy.

With the goal of deriving a valid measure of gut microbial assembly during infancy, our objective was to adapt the significance analysis of microarrays [SAM (13)] method to characterize and quantify changes to gut microbial abundance in the infant gut over time. This was done according to the common co-occurrence of early perinatal interventions, using fecal samples in full-term infants at 3 months and 1 year of age.

## Materials and Methods

Bacterial DNA was extracted from 166 paired fecal samples of full-term infants at age 3 months (mean age 3.1 ± 0.6 months, hereinafter termed 3 months) and 1 year (mean age 11.8 ± 0.8 months, hereinafter called 1 year) at the Winnipeg site of the CHILD (Canadian Healthy Infant Longitudinal Development) cohort (www.canadianchildstudy.ca) (14), using the QIAamp DNA Stool Mini Kit (Qiagen, Mississauga, ON, Canada), followed by MiSeq high-throughput 16S gene sequencing to identify individual microbes. Data on birth mode, infant antibiotic exposure (maternal intrapartum prophylaxis and/or postnatal infant treatment until 3 months of age) and breastfeeding status at 3 months were obtained from the birth record and from maternal report. Further details on the study population and data sources are described in Azad et al. (9).

Gut microbial taxa were typically quantified as measures of operational taxonomic unit (OTU) relative abundance from sequenced fecal samples. Infants were placed into mutually exclusive groups according to combinations of common perinatal exposures: birth mode, feeding status and antibiotic treatment. We applied the SAM method to capture change in OTU abundance from 3 to 12 months of age within each of the seven strata: (i) vaginal birth, breastfed by 3 months, no antibiotic exposure during birth or by 3 months of age (“less disturbed”); (ii) vaginal birth, breastfed with antibiotic exposure; (iii) vaginal birth, not breastfed with antibiotic exposure; (iv) vaginal birth, not breastfed, no antibiotic exposure; (v) elective cesarean (with antibiotic prophylaxis), breastfed; (vi) elective cesarean (with antibiotic prophylaxis), not breastfed, and (vii) emergency cesarean, breastfed. For the purposes of this study, elective cesarean was synonymous with scheduled cesarean section, and perinatal was defined as the time period from birth to 3 months. The small sample size for infants delivered by emergency cesarean and not breastfed was insufficient to run the permutations of the SAM method.

### Rationale for Adapting SAM to Quantify Longitudinal Changes in Gut Microbiota

The SAM method was introduced and extended to gene microarray studies for the purpose of better understanding mechanisms of disease. Similar to the analysis of gut microbial abundance in infants, a challenging analytical feature of DNA microarrays is that these data consist of a much larger number of features (*p*) than the sample size *N*, that is, *p* ≫ *N* (15). Smaller magnitude changes in OTU abundance can also create “noise” and interfere with finding true differences. By overcoming these underlying issues, especially where *p* ≫ *N*, SAM has enormous potential in advancing the longitudinal analysis of 16S sequenced data to characterize microbial community development over time.

### The SAM Method

Significance analysis of microarrays were proposed by Tusher et al. (13) and later extended and developed by Storey and Tibshirani (16). It is a popular analytical method that searches for statistically significant genes associated with a phenotype of interest in a microarray data set (17). We used SAM to identify changes in OTU abundance for paired data points at 3 months and 1 year of infant age. OTU relative abundance is correlated across time within the same infants, requiring a statistical method that accounts for this correlation. Implementing the SAM method for the computational analysis of our OTU data set, enabled us to perform repeated permutations which accounted for the correlation of OTU abundance measured at 3 months and 1 year.

Our data represent the paired measurements of OTU abundance at 3 months and 1 year in 166 infants who were followed prospectively. While testing thousands of microbial abundance changes within paired OTUs simultaneously, we needed to estimate an overall measure for the type 1 error in multiple hypothesis testing. Among the variety of methods to detect the type 1 error is the false discovery rate (FDR). FDR focuses on the proportion of falsely statistically significant differences (15). Storey and Tibshirani proposed the FDR correction for the SAM method (16), and details on its calculation are stated in SAM technical details by Chu et al. (18). The SAM output reported FDR values for each significantly significant change in OTU abundance. We chose a fixed FDR value of 0.1.

We also used employed Spearman correlation to assess the agreement between OTU changes and the maximum SAM score statistic for each perinatal group.

### Software Analysis Packages

We used R software, version 2.15.3 for executing the SAM method to generate SAM statistic plots for paired OTU abundance values within infants of each perinatal exposure group. A heat map to visually compare findings across exposure groups was also generated using “R.” SAS 9.3 and 9.4 were also used for data handling and manipulation.

## Results

We initially applied SAM to all 166 pairs of infants at 3 months and 1 year, and then to each of the 7 groups of infants stratified by perinatal co-exposures. As noted, the small sample size for infants delivered by emergency cesarean and not breastfed was insufficient to run the permutations of the SAM method. We excluded OTUs that had very low abundance in our data, specifically those with a 0 relative abundance. This approach was previously taken by de Steenhuijsen Piters et al. (19), using SAM to analyze upper respiratory tract microbiota in elderly pneumonia patients. The reason SAM excludes 0 value is because it applies a logarithmic transformation to reduce skewness. The resultant number of OTUs per perinatal group was approximately 1,000 as follows: (i) vaginal, breastfed, and no antibiotic use (1,124 OTUs), (ii) vaginal, breastfed, and antibiotic use (1,111 OTUs), (iii) vaginal, not breastfed, and antibiotic use (995 OTUs), (iv) vaginal, not breastfed, and no antibiotic use (1,094 OTUs), (v) elective CS, breastfed, and antibiotic use (1,069 OTUs), (vi) elective CS, not breastfed, and antibiotic use (918 OTUs), and (vii) emergency CS, breastfed, and antibiotic use (1,067 OTUs).

**Figure 1:**
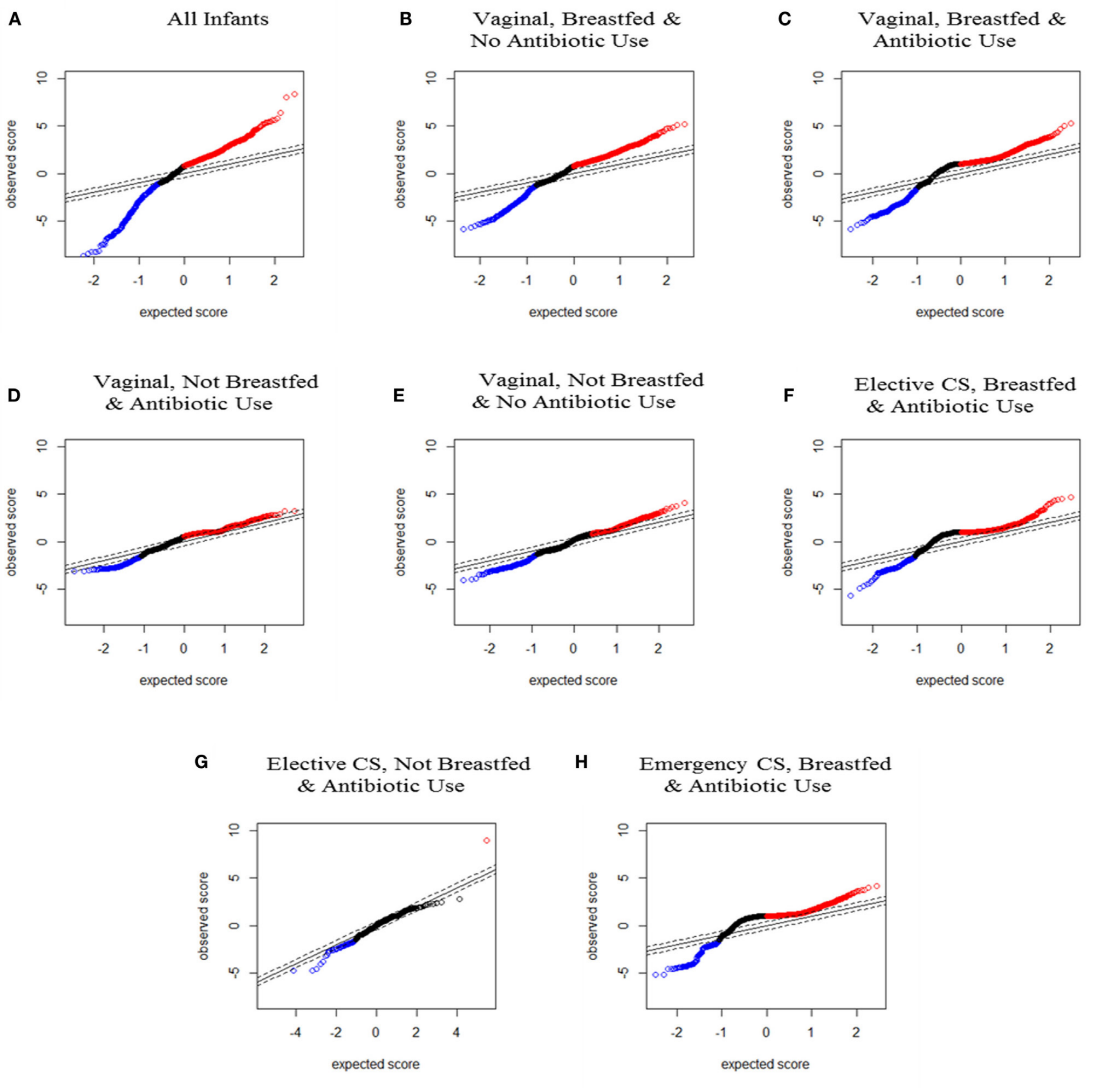
Significance analysis of microarrays (SAM) plots for all infants and for each seven perinatal exposure group from the paired SAM analysis. Red dots represent significantly increased and blue dots represent significantly decreased operational taxonomic units between 3–4 months and 1 year from SAM paired analysis. **(A)** For all infants (*n* = 166 pairs); **(B)** infants delivered vaginally, breastfed, and not exposed to antibiotics (*n* = 71 pairs); **(C)** infants delivered vaginally, breastfed, and exposed to antibiotics (*n* = 34 pairs); **(D)** infants delivered vaginally, not breastfed, and exposed to antibiotics (*n* = 8 pairs); **(E)** infants delivered vaginally, not breastfed, and not exposed to antibiotics (*n* = 17 pairs); **(F)** infants delivered by elective cesarean section, breastfed, and exposed to antibiotics (*n* = 13 pairs); **(G)** infants delivered by elective cesarean section, not breastfed, and exposed to antibiotics (*n* = 3 pairs), and **(H)** infants delivered by emergency cesarean section, breastfed, and exposed to antibiotics (*n* = 16 pairs).

Data were visually represented as plots of SAM scores to show statistically significant changes in OTU relative abundance from 3 months to 1 year. The SAM score denotes the statistical significance of each OTU change and can be graphed according to the observed and expected score (see Figure 1). The SAM score plots of the lines for observed versus expected SAM scores allowed visual comparison of the deviation in OTU abundance changes, namely, which longitudinal changes were greater or less than expected, and which were not statistically different. Creating an SAM plot for each perinatal exposure grouping also enabled visual comparison of the shape of observed SAM score lines across exposure groups and against the gold standard of infants delivered vaginally, breastfed and not exposed to antibiotics, labeled the “less disturbed group.” All perinatal exposures produced some deviation from the less disturbed group but the shape of the SAM score line for infants not breastfed was the most deviant.

As reported in Table 1 and Table S1 in Supplementary Material, the SAM score can also be quantified according to the total number and percent of changes to relative abundance by type (increasing, decreasing, and no change) for all OTUs and for OTUs within their phylogenetic groups (phyla). A large number of OTUs did not show significant changes to abundance over the 3- to 12-month time period.

**Table 1:** Statistically significantly increased, decreased, and unchanged microbial [operational taxonomic unit (OTU)] abundance from 3 months to 1 year per infant perinatal exposure group.

Number of OTU changes in abundance from 3 months to 1 year by infant exposure group, % (*n*)	Any change to OTU	Increased abundance	Decreased abundance	Unchanged abundance
Vaginal, breastfed, and no antibiotic use (infants = 71 data pairs, total *N* = 1,124 OTU)	63.3% (712)	47.7% (536)	15.7% (176)	36.7% (412)
Vaginal, breastfed, and antibiotic use (infants = 34 data pairs, total *N* = 1,111 OTU)	68.1% (757)	50.04% (556)	18.1% (201)	31.9% (354)
Vaginal, not breastfed, and no antibiotic use (infants = 17 data pairs, total *N* = 1,094 OTU)	32.2% (352)	12.1% (132)	20.1% (220)	67.8% (742)
Vaginal, not breastfed, and antibiotic use (infants = 8 data pairs, total *N* = 995 OTU)	57.4% (571)	47% (468)	10.4% (103)	42.6% (424)
Elective CS, breastfed, and antibiotic use (infants = 13 data pairs, total *N* = 1,069 OTU)	57.8% (618)	50% (535)	7.80% (83)	42.2% (451)
Elective CS, not breastfed, and antibiotic use (infants = 3 data pairs, total *N* = 918 OTU)	0.65% (6)	0.11% (1)	0.54% (5)	99.35% (912)
Emergency CS, breastfed, and antibiotic use (infants = 16 data pairs, total *N* = 1,067 OTU)	56.4% (602)	49.7% (530)	6.7% (72)	43.6% (465)
Emergency CS, no breastfed, and antibiotic use	X	X	X	X

*Significance analysis of microarrays could not be executed for infants born by emergency cesarean and not breastfed due to the small number of infants in that group*.

### Phylum Level Changes According to Perinatal Exposure Groups

Infants delivered vaginally, with no antibiotic exposure and breastfeeding by 3 months demonstrated a 48% (28% Firmicutes and 11% Bacteroidetes) increase and a 16% (3% Proteobacteria and 9% Firmicutes) decline in OTU abundance over the next 9–11 months. More than one-third of OTUs in these “less disturbed” infants did not change in abundance. Increases (28.5% Firmicutes and 12% Bacteroidetes) or decreases (3.5% Proteobacteria and 10% Firmicutes) in OTU abundance were slightly more common when vaginally delivered, breastfed infants were exposed to antibiotics by 3 months of age.

Exclusive formula feeding combined with antibiotic exposure after a vaginal birth resulted in an OTU rise similar in proportion to “less disturbed” infants (26% Firmicutes and 11% Bacteroidetes), but much fewer OTUs (10%) declined in abundance (2% Proteobacteria), such that 43% of OTU abundance was unchanged.

The proportion of unchanged OTU abundance rose further to 68% in vaginally delivered and formula-fed infants who had not been exposed to antibiotics. At 20%, the decline in total OTU abundance in the gut microbiota of these infants was the highest (4% Proteobacteria and 12% Firmicutes) and notably, for Bacteroidetes (4%). Only 12% of OTUs (7% Firmicutes and 3% Bacteroidetes) had become more abundant. In contrast to the other vaginally born groups, the Actinobacteria (bifidobacteria-like microbes) rose by <1%.

Infants born by elective or emergency cesarean (and exposed to antibiotics) who were still being breastfed by 3 months of age had a similar profile, seen as a 50% (28% Firmicutes and 12% Bacteroidetes) enrichment and at 7–8% (<1% Proteobacteria), a much lower decline in OTU abundance than vaginally delivered infants. Infants born by elective cesarean and not breastfed by 3 months had the most deviant profile, with almost no increases or decreases in OTU abundance; although based on a comparable number of OTUs to other groups, these results were in a small number of infants. Similar to vaginally delivered infants not breastfed or exposed to antibiotics, these infants had the highest percent of unchanged OTU abundance in the Proteobacteria phylum. Also in these 2 groups of infants, a greater proportion of Bacteroidetes OTUs were unchanged and much fewer Bacteroidetes species increased their abundance.

### Family Level Changes According to the SAM Score

According to the maximum SAM score for OTU increase (or decrease), perinatal exposure groups were ranked from highest to lowest scores as follows (Table 2): breastfed after vaginal birth with/without antibiotic treatment, breastfed after cesarean delivery, and not breastfed after vaginal birth with/without antibiotics. At the family level, percent increases to the abundance of *Veillonellaceae* and of *Clostridiaceae* were highly positively corrected with the SAM score. Infants exclusively fed formula at 3 months after a vaginal birth had the lowest rates of rising abundance of these two Firmicutes families. Enrichment (or depletion) of the *Bacteroidaceae* or *Enterobacteriaceae* did not follow the same pattern by perinatal co-exposure (data not shown). However, the ratio of *Rikenellaceae* to *Bacteroidaceae* within depleted OTUs was highly correlated with the SAM score.

**Table 2:** Correlation between maximum significance analysis of microarrays (SAM) score per perinatal group and select operational taxonomic unit (OTU) changes at the family level.

↑ = Increased ↓ = Decreased ↔ = Unchanged	Maximum^a^ SAM score for OTU increases	% OTUs = ↑ *Veillonellaceae*	% OTUs = ↑ *Clostridiaceae*	% OTUs = ↔ *Ruminococcaceae*	% OTUs = ↔ *Bacteroidaceae*	*Rikenellaceae: Bacteroidaceae* ratio of ↓ OTUs	% OTUs = ↔ *Enterobacteriaceae*	% OTUs = ↔ *Alcaligenaceae*	*Enterobacteriaceae: Alcaligenaceae* ratio of ↔ OTUs
Vaginal, breastfed, and no antibiotic use (infants = 71 data pairs, total *N* = 1,124 OTU)	5.2	3.1	2.4	4.4	5.4	0.19	4.1	0.53	7.7

Vaginal, breastfed, and antibiotic use (infants = 34 data pairs, total *N* = 1,111 OTU)	5.3	2.9	2.6	3.9	4.4	0.15	3.2	0.63	5.1

Elective CS, breastfed, and antibiotic use (infants = 13 data pairs, total *N* = 1,069 OTU)	4.5	2.9	2.0	4.9	6.2	0.20	5.4	0.66	8.3

Emergency CS, breastfed, and antibiotic use (infants = 16 data pairs, total *N* = 1,067 OTU)	4.1	2.6	1.8	5.9	9.4	0.18	6.8	0.84	8.0

Vaginal, not breastfed, and no antibiotic use (infants = 17 data pairs, total *N* = 1,094 OTU)	4.0	0.9	0.6	8.2	7.1	0.23	7.2	0.55	13.2

Vaginal, not breastfed, and antibiotic use (infants = 8 data pairs, total *N* = 995 OTU)	3.3	2.4	1.8	6.4	11.1	0.33	5.7	0.70	8.1

Spearman correlation between SAM score and column % or ratios		0.84, *p* < 0.04	0.90, *p* < 0.02	−0.94, *p* < 0.005	−0.94, *p* < 0.005	−0.83, *p* = 0.04	−0.83, *p* = 0.04	−0.43, *p* = 0.4	−0.71, *p* = 0.11

*^a^Similar correlations were obtained for the maximum SAM score for OTU decreases*.

Regarding OTUs that did not change in abundance (Table 2), high negative correlations with the SAM score were obtained for the fecal abundance of *Ruminococcaceae, Bacteroidaceae*, and *Enterobacteriaceae*. Infants born vaginally and not breastfed at age 3 months had the lowest percentages of unchanging *Alcaligenaceae*; there was a borderline inverse correlation between the SAM score and the *Enterobacteriaceae* to *Alcaligenaceae* ratio. Switching the rank order of elective versus emergency CS improved the statistical significance of this correlation (*r* = −0.77, *p* = 0.07).

**Figure 2:**
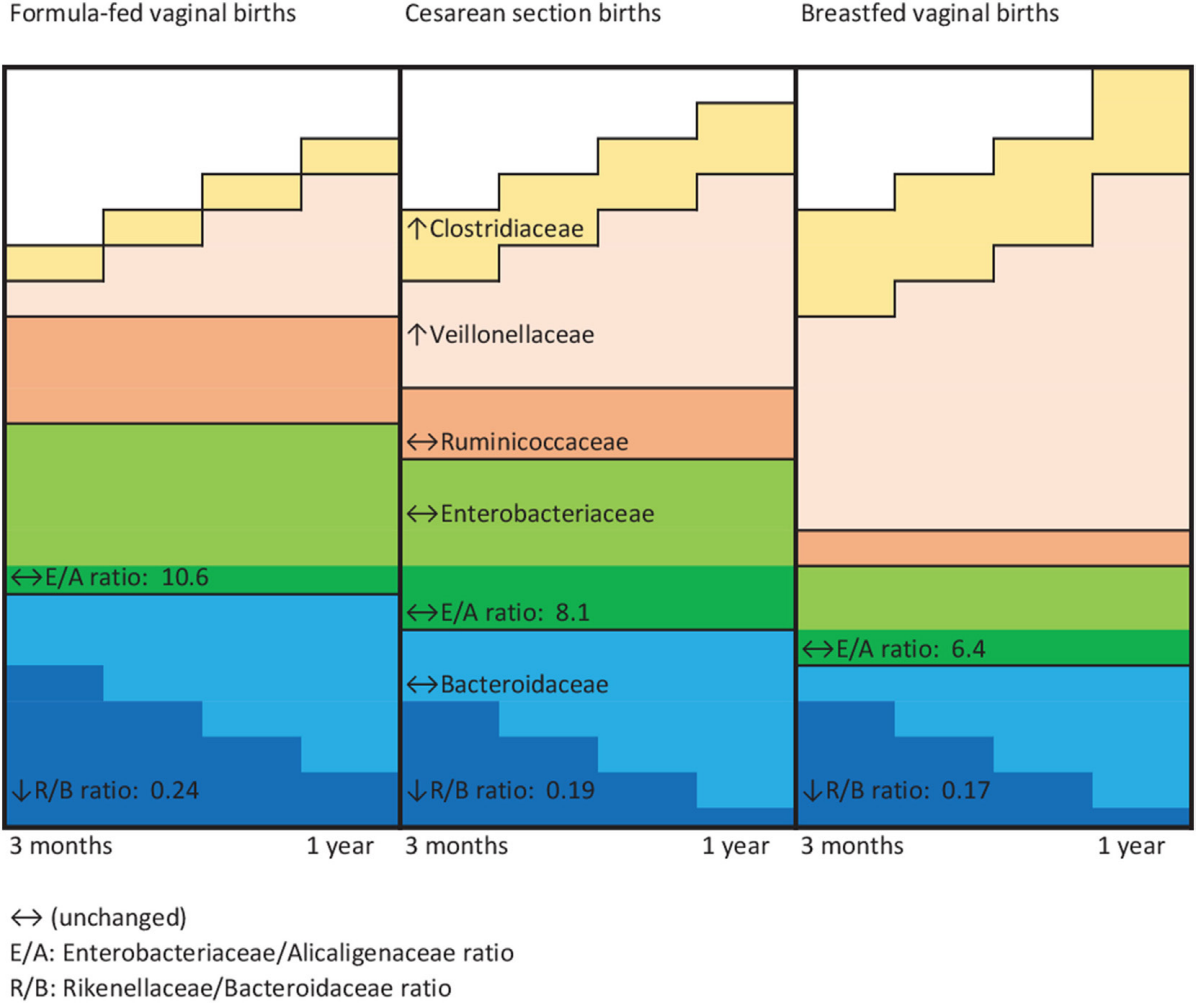
Schematic representation of microbial family changes by perinatal group. ↔, Unchanged; E/A, *Enterobacteriaceae*/*Alcaligenaceae* ratio; R/B, *Rikenellaceae*/*Bacteroidaceae* ratio.

Figure 2 is a conceptual representation of the aforementioned family level variations by perinatal co-exposure. From 3 months to 1 year of age, the gut microbiota of infant vaginally born and not breastfed became less enriched with family *Veillonellaceae* and family *Clostridiaceae*, and exhibited a greater decline in *Rikenellaceae* relative to *Bacteroidaceae* OTUs. Their fecal microbiota were most likely to show unchanging levels of *Bacteroidaceae, Enterobacteriaceae*, and *Ruminococcaceae*. Cesarean-delivered breastfed infants exhibited these microbial changes to an extent intermediate to that of vaginally born, not breastfed infants and vaginally born, breastfed infants.

### Genus Level Changes According to the SAM Score

**Figure 3:**
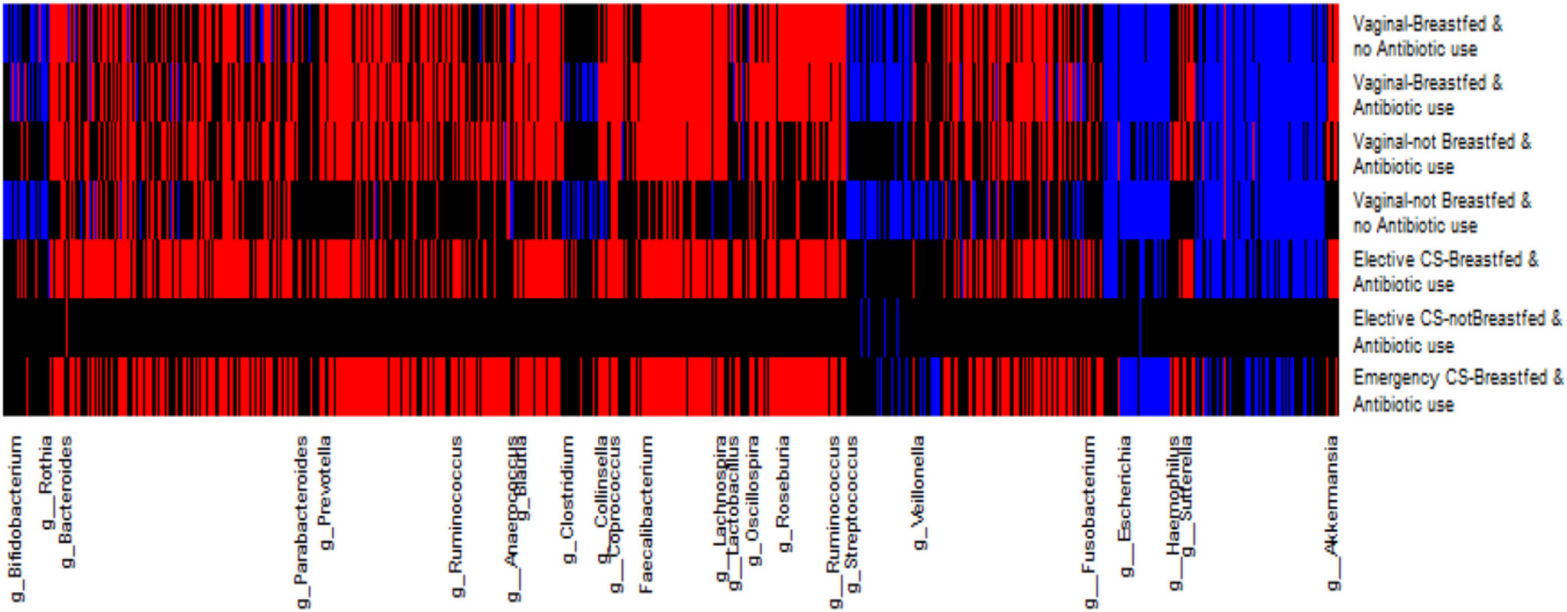
Heat map for each operational taxonomic unit (OTU) change in relative abundance grouped at the genus level by perinatal exposure group. The relative abundance of red-colored OTUs increased and blue colored OTUs decreased from 3 months to 1 year; black colored OTUs were unchanged over this time period.

As many others have done, we created a heat map of SAM scores by OTU genus category and perinatal exposure group to visually differentiate across perinatal exposures, the degree of OTU abundance change at the genus level (Figure 3). The magnitude of the SAM statistic was used to characterize the degree of change from the most depleted to the most enriched in OTU abundance. Similar to the SAM plots, infants not breastfed (vaginally born or delivered by elective cesarean) exhibited the greatest deviation of OTU depletion, enrichment, or no change at the genus level from the less disturbed group. Infants born by emergency cesarean and breastfed showed more similar changes to the gold standard of vaginal delivery, breastfeeding, and no antibiotic exposure.

Ranked by the magnitude of their SAM score in Tables 3 and 4, we list the top 10 OTUs that rose and declined in relative abundance, respectively. For the gold standard trajectory of vaginally born, breasted infants not exposed to antibiotics, statistically significant increases in relative abundance were seen for lactate producers (*Lactobacillus* and *Streptococcus*) and lactate utilizers, *Veillonella, Roseburia, Faecalibacterium*, and other *Lachnospiraceae*. Several species of the *Bacteroidaceae* and *Ruminococcaceae* also increased in abundance. Reductions in abundance were also seen for microbes in these families, along with members of the *Enterobacteriaceae*. Uniquely in these infants, *Fusobacterium* was among the top 10 taxa that increased in abundance.

**Table 3:** Top 10 statistically significant increases to operational taxonomic unit (OTU) abundance from 3 months to 1 year of infant age at the genus and species level.

Vaginal, breastfed, and no antibiotic use		Vaginal, breastfed, and antibiotic use		Vaginal, not breastfed, and no antibiotic use		Vaginal, not breastfed, and antibiotic use		Elective CS, breastfed, and antibiotic use		Elective CS not breastfed and antibiotic use		Emergency CS, breastfed, antibiotic use	
↑ OTU	Significance analysis of microarrays (SAM) score	↑ OTU	SAM Score	↑ OTU	SAM Score	↑ OTU	SAM Score	↑ OTU	SAM Score	↑ OTU	SAM Score	↑ OTU	SAM Score
*Veillonella* sp.	5.2	*Bacteroides caccae*	5.3	Unclassified *Enterobacteriaceae* sp.	4.0	*Bacteroides ovatus*	3.3	Unclassified Clostridia	4.7	*Ruminococcus* sp.	9.0	*Anaerostipes* sp.	4.1

*Roseburia* sp.	5.1	Unclassified *Peptostreptococcaceae* sp.	5.0	*Bacteroides* sp.	3.7	Unclassified *Enterobacteriaceae* sp.	3.2	*Bacteroides* sp.	4.5			*Streptococcus* sp.	4.0

*Fusobacterium* sp.	4.9	*Veillonella* sp.	4.6	*Oscillospira* sp.	3.6	*Citrobacter* sp.	2.9	Unclassified Clostridia	4.5			*Ruminococcus gnavus*	3.7

*Streptococcus* sp.	4.8	Unclassified *Lachnospiraceae* sp.	4.3	Unclassified *Lachnospiraceae* sp.	3.4	*Bacteroides* sp.	2.8	Unclassified *Lachnospiraceae* sp.	4.3			Unclassified *Lachnospiraceae* sp.	3.7

Unclassified *Ruminococcaceae* sp.	4.8	Unclassified *Ruminococcaceae* sp.	4.2	*Bacteroides uniformis*	3.4	*Actinomyces* sp.	2.8	*Citrobacter* sp.	4.1			*Parabacteroides* sp.	3.6

*g__Lactobacillus* *f__Lactobacillaceae*	4.7	*g__Streptococcus* *f__Streptococcaceae*	4.2	*g__Ruminococcus* *f__Lachnospiraceae*	3.3	*g__Streptococcus* *f__Streptococcaceae*	2.8	*g__Actinomyces* *f__Actinomycetaceae*	4.0			*g__Parabacteroides* *f__Porphyromonadaceae*	3.6

*f__Lachnospiraceae* Unclassified genus and species	4.4	*f__Coriobacteriaceae* Unclassified genus and species	4.0	*f__Lachnospiraceae* Unclassified genus and species	3.2	*f__Lachnospiraceae* Unclassified genus and species	2.8	*g__Streptococcus* *f__Streptococcaceae*	3.9			*g__Atopobium* *f__Coriobacteriaceae*	3.6

*scaccae* *g__Bacteroides*	4.4	*f__Lachnospiraceae* Unclassified genus and species	3.9	*g__Bacteroides* *f__Bacteroidaceae*	3.1	*f__Lachnospiraceae* Unclassified genus and species	2.7	*f__Ruminococcaceae* Unclassified genus and species	3.9			*g__Bifidobacterium* *f__Bifidobacteriaceae*	3.4

*f__Lachnospiraceae* Unclassified genus and species	4.3	*f__Enterobacteriaceae* Unclassified genus and species	3.9	*g__Veillonella* *f__Veillonellaceae*	3.1	*f__Ruminococcaceae* Unclassified genus and species	2.7	*sdispar* *g__Veillonella*	3.7			*sproducta* *g__Blautia*	3.4

*g__Faecalibacterium* *f__Ruminococcaceae*	4.3	*g__Lachnospira* *f__Lachnospiraceae*	3.8	*g__Blautia* *f__Lachnospiraceae*	3.1	*f__Enterobacteriaceae* Unclassified genus and species	2.7	*f__Enterobacteriaceae* Unclassified genus and species	3.6			*s__gnavus* *g__Ruminococcus*	3.4

**Table 4:** Top 10 statistically significant decreases in operational taxonomic unit (OTU) abundance from 3 months to 1 year of infant age at the genus and species level.

Vaginal, breastfed, and no antibiotic use		Vaginal, breastfed, and antibiotic use		Vaginal, not breastfed, and no antibiotic use		Vaginal, not breastfed, and antibiotic use		Elective CS, breastfed, and antibiotic use		Elective CS, not breastfed, and antibiotic use		Emergency CS, breastfed, and antibiotic use	
↓ OTU	Significance analysis of microarrays (SAM) score	↓ OTU	SAM Score	↓OTU	SAM Score	↓ OTU	SAM Score	↓OTU	SAM Score	↓OTU	SAM Score	↓OTU	SAM Score
*Bacteroides* sp.	−5.9	Unclassified *Lachnospiraceae* sp.	−5.9	*Bacteroides caccae*	−4.0	*Escherichia* sp.	−3.1	*Parabacteroides distasonis*	−5.7	*Bacteroides* sp.	−10.1	Unclassified *Ruminococcaceae* sp.	−5.2

*Faecalibacterium prausnitzii*	−5.7	*Bacteroides* sp.	−5.4	Unclassified *Enterobacteriaceae* sp.	−4.0	*Faecalibacterium* sp.	−3.1	*B. caccae*	−4.9	*Veillonella dispar*	−4.8	*Bacteroides* sp.	−5.1

Unclassified *Ruminococcaceae* sp.	−5.5	*P. distasonis*	−5.2	*F. prausnitzii*	−3.9	*P. distasonis*	−3.0	*Lactobacillus reuteri*	−4.6	*Akkermansia muciniphila*	−4.8	*Bacteroides ovatus*	−4.6

*Streptococcus* sp.	−5.3	*Streptococcus* sp.	−5.2	*Escherichia* sp.	−3.5	Unclassified Alphaproteobacteria	−3.0	*Roseburia* sp.	−4.4	Unclassified *Enterobacteriaceae* sp.	−4.6	*Butyricimonas* sp.	−4.5

Unclassified *Enterobacteriaceae* sp.	−5.3	*F. prausnitzii*	−5.0	*Roseburia* sp.	−3.5	*Oscillospira* sp.	−3.0	Unclassified *Lachnospiraceae* sp.	−4.4	*Anaerococcus hydrogenalis*	−4.1	*Prevotella copri*	−4.5

*f__Ruminococcaceae* Unclassified genus and species	−5.2	*f__Enterobacteriaceae* Unclassified genus and species	−4.8	*g__Oscillospira* *f__Ruminococcaceae*	−3.5	*g__Enterococcus* *f__Enterococcaceae*	−3.0	*g__Faecalibacterium* *f__Ruminococcaceae*	−4.2			*f__Lachnospiraceae* Unclassified genus and species	−4.1

*f__Peptostreptococcac* Unclassified genus and species	−5.2	*s__ovatus* *g__Bacteroides*	−4.7	*s__fragilis* *g__Bacteroides*	−3.3	*f__Ruminococcaceae* Unclassified genus and species	−3.0	*g__Escherichia* *f__Enterobacteriaceae*	−4.1			*g__Bifidobacterium* *f__Bifidobacteriaceae*	−4.4

*g__Lactobacillus* *f__Lactobacillaceae*	−5.1	*g__Butyricimonas* *f__Odoribacteraceae*	−4.6	*s__fragilis* *g__Bacteroides*	−3.2	*s__copri* *g__Prevotella*	−2.9	*f__Ruminococcaceae* Unclassified genus and species	−3.9			*f__Ruminococcaceae* Unclassified genus and species	−4.4

*f__Rikenellaceae* Unclassified genus and species	−5.1	*f__Ruminococcaceae* Unclassified genus and species	−4.6	*f__Ruminococcaceae* Unclassified genus and species	−3.2	*s__fragilisg__Bacteroides*	−2.9	*f__Lachnospiraceae* Unclassified genus and species	−3.7			*s__fragilisg__Bacteroides*	−4.4

*g__Blautia* *f__Lachnospiraceae*	−4.9	*s__gnavus* *g__Ruminococcus*	−4.5	*g__Bacteroides* *f__Bacteroidaceae*	−3.2	*g__Eubacterium* *f__Erysipelotrichaceae*	−2.9	*f__Lachnospiraceae* Unclassified genus and species	−3.6			*f__Lactobacillaceae* Unclassified genus and species	−4.6

Maternal intrapartum antibiotic prophylaxis and/or infant antibiotic treatment slightly modified this microbial succession, in that the most statistically significant changes in abundance were seen as enrichment with genus *Bacteroides* and the peptostreptococci. However, the Enterobacteria also became more abundant and reductions in several species of the Bacteroidetes phylum were evident.

The absence of breastfeeding by 3 months of age in vaginally born, antibiotic-free infants also modified this succession. Their fecal samples became enriched with several families of the Enterobacteria and depleted in members of the Bacteroidetes phylum. *Oscillospira* of the ruminococci also declined in abundance. The combined effect of antibiotic exposure and absence of breastfeeding resulted in further changes among vaginally born infants. Enterobacteria increased the most in the fecal samples of these infants and several species of Bacteroidetes became less abundant.

The impact of cesarean delivery on taxon change depended on the delivery type. Under the scenario of emergency cesarean and breastfeeding by 3 months, significant increases in abundance were seen for streptococci and bifidobacteria, as well as for some *Lachnospiraceae*. Species in the Bacteroidetes phylum for the most part, declined in abundance, including members of several different families: *Bacteroidaceae, Prevotellaceae*, Odoribacteracea (*Butyricimonas*), and *Porphyromonadaceae* (*Parabacteroides*). Unlike for other infant groups, Enterobacteria were not among the top 10 of OTU abundance reductions or increases.

Infants delivered by elective cesarean who were breastfed exhibited a different trajectory of microbial change. In these infants, two species of clostridia and of Enterobacteria become more abundant. The lactobacilli and *Bacteroides* species declined in abundance, as did several species of lactate utilizers (*Lachnospira, Roseburia*, and *Faecalibacterium*). Few changes to microbial composition from 3 months to 1 year were observed following elective cesarean in the absence of breastfeeding. Only the ruminococci rose in abundance among these infants, whereas genus *Akkermansia* and *Bacteroides* were depleted.

With the exception of the less disturbed group of infants, all breastfed infants exhibited increases to the abundance of bifidobacteria or related species in the Actinobacteria phylum. This enrichment was seen to a much lesser extent in the absence of breastfeeding following vaginal or elective cesarean delivery.

## Discussion

In a population of 166 full-term infants, the SAM method proved useful in differentiating statistically significant change in gut microbial abundance between 3 months and 1 year of age according to common combinations of birth mode, antibiotic exposure and breastfeeding status. Breastfed infants who were born vaginally and not exposed to antibiotics within 3 months after birth, exhibited a unique trajectory of gut microbial development in later infancy. Their gut microbiota most often showed significant increases to the abundance of lactate producers, such as lactobacilli and streptococci, and of several lactate utilizers belonging to genus *Veillonella*, genus *Roseburia*, and genus *Faecalibacterium* of the Firmicutes phylum. Lactate utilizers provide the host with important short-chain fatty acid metabolites, shown to prevent allergic disease in experimental models (20). Overall, the less disturbed gut microbiota in these infants exhibited the highest abundance increases in the *Veillonellaceae* and *Clostridiaceae* families. In contrast, vaginally delivered infants not breastfed had the lowest rates of enrichment with *Veillonellaceae* and *Clostridiaceae*. *Veillonella* species have also been found to be depleted among food-sensitized infants at 14 months of age in the Chen et al. study (10).

As documented by many (1–7), commonly observed patterns of microbial succession over the first year of life are declining abundance of Proteobacteria, the pioneer inhabitants of the gut, and a gradual rise in colonization with members of the Firmicutes and Bacteroidetes phyla. Indeed, this trajectory was observed in the less disturbed microbiome group and other infants in our study. It is informative then, to learn which perinatal co-exposures caused a deviation from this trajectory. We found cesarean delivery (with antibiotic exposure) or exclusive formula feeding after a vaginal birth with antibiotic exposure did not yield the typical decline in Proteobacterial abundance in gut microbiota after age 3 months. Unchanging abundance of *Enterobacteriaceae* between 3 months and 1 year of age was highest among formula-fed infants, who had the lowest SAM scores, indicating the least statistically significant OTU increases or decreases within the perinatal groups. In our previous study, elevated enterobacterial colonization at 3 or 12 months of age was found to be a risk factor for food sensitization in the CHILD birth cohort; at 6 months of age, these Gram-negative bacteria also predict future adiposity (5, 9).

Consistent with initial depletion of Bacteroidetes after cesarean birth (4, 7) our cesarean breastfed infants experienced higher rates of increase to Bacteroidetes species abundance than vaginally delivered infants. Infants not breastfed after a vaginal birth without antibiotic exposure or after an elective cesarean were more likely to show unchanging fecal abundance of Bacteroidetes species. On the other hand, antibiotic exposure in vaginally born, not breastfed infants yielded similar increases in Bacteroidetes abundance to the less disturbed group, but concurrently fewer changes to *Enterobacteriaceae*. In this group of infants, as well as in infants delivered by elective cesarean, a higher percentage of the beta-Proteobacteria, namely, *Alcaligenaceae* (or genus *Sutterella*), also did not change in abundance over the 3- to 12-month period. As noted earlier, beta-Proteobacteria increase their abundance in later infancy following the introduction of solid foods. In our previous paper, *Alcaligenaceae* were depleted in gut microbiota at 1 year among food-sensitized infants (9).

Unique changes to microbial composition across perinatal co-exposures were also evident. Only in the less disturbed microbiota group did the *Fusobacterium* microbe appear in the top 10 most enriched species. A common member of the oral microbiome, *Fusobacterium*, has been recently detected in the oral cavity of 6- and 12-month infants following the eruption of teeth (21). Further, increases to the abundance of genus *Ruminococcus* were the sole change in elective cesarean in the absence of breastfeeding. Within most other exposure groups, ruminococcal species both increased and decreased in their abundance. Yet, unchanging abundance of the family *Ruminococcaceae* was a feature of the gut microbiota of vaginally born infants not breastfed. Ruminococci stimulate the production and degradation of mucin, required to maintain an intact microbiota-mucin barrier (22). They predominate in formula-fed infants; in breastfed infants, their concentrations depend on the oligosaccharide content of breast milk (23, 24). As fiber degraders (25), they become more plentiful after infant weaning off the breast (26). Previously, we observed a strong cross-sectional association between low levels of *Ruminococcaceae* and food sensitization at age of 1 year (9).

The SAM method identified statistically significant changes at the family, genus or species level that were counter to the overall trend for a phylum. This also occurred in infants with a less disturbed gut microbiome, in whom reductions in the Bacteroidetes phylum were seen, with *Bacteroides* and *Rikenella* species appearing in the top 10 statistically significant reductions. What differentiated these changes from other infants was the ratio of *Rikenellaceae* to *Bacteroidaceae* species within the Bacteroidetes phylum that declined in abundance. The ratio was largest among infants who were born vaginally but not breastfed at 3 months of age. Cesarean-delivered infants who were breastfed by 3 months of age had fewer reductions in Bacteroidetes species than the less disturbed group, but these reductions involved several families: *Bacteroidaceae, Prevotellaceae, Odoribacteracea* (*Butyricimonas*), and *Porphyromonadaceae* (*Parabacteroides)*. Jakobsson also reported reductions in microbial diversity within the Bacteroidetes phylum for cesarean-sectioned infants against a backdrop of rising abundance with infant age (7).

Employing the SAM method afforded several strengths to our analyses. As shown in this study, heat maps are visually effective in characterizing changes to microbial composition during infancy. Indeed, hundreds of species changes were evident within the less disturbed gut microbiome. Correlation or *t*-test methods to determine the statistical significance of these changes is computationally intensive and subject to type 1 error. The SAM method permitted simultaneous comparisons among a large number OTUs that were FDR adjusted to identify which species changes in abundance were statistically significant. It also addressed the small variability inherent in microbial abundance during infancy through a modification of the *t*-test statistic. Within these parameters, the SAM method revealed that the fewest changes in microbial abundance occurred in infants delivered by elective cesarean and not breastfed, followed by infants that were not breastfed after vaginal delivery.

On the other hand, limitations to the SAM method included our inability to adjust for individual confounding factors and to analyze sets of multiple, correlated measures, such as sets of OTUs at higher taxonomic categories sharing a common biological function. The first limitation is why we grouped infants according to seven combinations of common birth and feeding exposures. While this suited our primary research question on the influence of concurrent early life exposures, we were unable to compare across infant groups or to test the independence of one exposure over another. Regarding the second limitation, we tested age-related change in abundance within each OTU, then collapsed findings at the phylum, family or genus level. As shown in other studies we have conducted, some statistical associations between health outcomes and changes to gut microbiota are seen at higher taxonomic levels, which we did not ascertain. Finally, these analyses were conducted within the Winnipeg site of the CHILD cohort, with the potential for slight differences in the co-occurrence of perinatal exposures from the three other CHILD study sites.

To conclude, we assessed microbial abundance changes from early to late infancy in over 1,000 species found in the gut microbiota of infants to characterize usual trajectories of microbiome development following common perinatal co-exposures. This was accomplished with the SAM permutation-based method to simultaneously execute a large number of tests for statistical significance, taking into account correlation in OTU abundance over infant age and adjusting for multiple comparisons. The SAM method has potential application in determining the statistical significance of age-related changes to microbial metabolites or metabolic pathways. With an increased sample size and the development of statistical methods to determine differences between groups or individual infants, quantifying early life changes to gut microbial taxon, metabolite or metabolic pathways with infant age has tremendous value in predicting health outcomes. Ultimately, the collective assessment of these microbial parameters will contribute to our understanding on what is normal gut microbial development in infants and what deviations from normal development predict future disease.

## Child Study Investigators

CHILD Study Investigators: **Anand S. S.**, McMaster University; **Azad M. B.**, University of Manitoba; **Becker A. B.**, University of Manitoba; **Befus A. D.**, University of Alberta; **Brauer M.**, University of British Columbia; **Brook J. R.**, University of Toronto; **Chen E.**, Northwestern University, Chicago; **Cyr M. M.**, McMaster University; **Daley D.**, University of British Columbia; **Dell S. D.**, The Hospital for Sick Children and University of Toronto; **Denburg J. A.**, McMaster University; **Duan Q. L.**, Queen’s University; **Eiwegger T.**, The Hospital for Sick Children and University of Toronto; **Grasemann H.**, The Hospital for Sick Children and University of Toronto; **HayGlass K.**, University of Manitoba; **Hegele R. G.**, The Hospital for Sick Children and University of Toronto; **Holness D. L.**, University of Toronto; **Hystad P.**, Oregon State University; **Kobor M.**, University of British Columbia; **Kollmann T. R.**, University of British Columbia; **Kozyrskyj A. L.**, University of Alberta; **Laprise C.**, Université du Québec à Chicoutimi; **Lou W. Y. W.**, University of Toronto; **Macri J.**, McMaster University; **Mandhane P. J.**, University of Alberta; **Miller G.**, Northwestern University, Chicago; **Moraes T. J.**, The Hospital for Sick Children and University of Toronto; **Paré P.**, University of British Columbia; **Ramsey C.**, University of Manitoba; **Ratjen F.**, The Hospital for Sick Children and University of Toronto; **Sandford A.**, University of British Columbia; **Scott J.**, University of Toronto; **Scott J. A.**, University of Toronto; **Sears M. R.** (Founding Director), McMaster University; **Silverman F.**, University of Toronto; **Simons E.**, University of Manitoba; **Subbarao P.** (Director), The Hospital for Sick Children and University of Toronto; **Takaro T.**, Simon Fraser University; **Tebbutt S. J.**, University of British Columbia; **To T.**, The Hospital for Sick Children and University of Toronto; **Turvey S. E.** (codirector), University of British Columbia.

## Ethics Statement

This study was approved by the Human Research Ethics Boards of the University of Alberta and the University of Manitoba. Written informed consent was obtained from all mothers.

## Author Contributions

FY performed the SAM statistical analysis, prepared the tables and figures, and wrote the first draft of the manuscript. HT completed the correlation analyses and related tables. ID identified the statistical methods and supervised the statistical analysis and interpretation of the results. AK conceived the study design and obtained funding, interpreted results, and wrote the final version of the manuscript. The microbiota profiling was completed by TK, DG, and JS. JS, DG, AB, PM, PS, ST, CF, RC, and MS helped obtain funding, advised on the study design, and coordinated data collection. All the authors provided critical comments on the manuscript content and approved the final version of the manuscript.

## Conflict of Interest Statement

The authors declare that the research was conducted in the absence of any commercial or financial relationships that could be construed as a potential conflict of interest.
