# Supplementary material for: Cesarean Section, Formula Feeding, and Infant Antibiotic Exposure: Separate and Combined Impacts on Gut Microbial Changes in Later Infancy
Source: Front Pediatr. 2017 Sep 26;5:200. doi: 10.3389/fped.2017.00200 (PMC5622971; doi:10.3389/fped.2017.00200)
Supplement: Supplementary file 1 [file table_1.pdf]

Table S1: Increases and decreases to microbial (OTU) abundance within perinatal exposure groups from 3 months to 1 year of infant age at the phylum level

Phyla	Vaginal Breastfed & No Antibiotic use (infants=71 pairs, total 1124)			Vaginal Breastfed & Antibiotic use (infants=34 pairs, Total 1111)			Vaginal Not Breastfed & Antibiotic use (infants=8 pairs, Total 995)			Vaginal Not Breastfed & No Antibiotic use (infants=17 pairs, Total 1094)			Elective CS Breastfed & Antibiotic-use (infants=13 pairs, Total 1069)			Elective CS Not Breastfed & Antibiotic-use (infants=3 pairs, Total 918)			Emergency CS Breastfed Antibiotic-use (infants=16 pairs, total 1067)		
Increase = ↑<br>Decrease = ↓<br>Unchanged = ↔	↑	↓	↔	↑	↓	↔	↑	↓	↔	↑	↓	↔	↑	↓	↔	↑	↓	↔	↑	↓	↔
p__Actinobacteria	1.5% (17)	0.3% (3)	1.6% (13)	1.8% (20)	0.4% (4)	0.8% (9)	1.8% (18)	0.3% (3)	1.0% (10)	0.6%(7)	0.3% (3)	2.1% (23)	1.6% (17)	0.1% (1)	1.4% (15)	0	0	3.2% (29)	1.9% (20)	0.2% (2)	1.0% (11)
p__Bacteroidetes	10.9% (123)	3.2% (36)	8.4% (94)	11.5% (128)	3.7% (41)	7.5% (83)	11.1% (110)	2.2% (22)	9.2% (92)	2.7% (29)	4.0% (44)	15.9% (174)	12.0% (128)	1.3% (14)	8.6% (92)	0	0.1% (1)	22.2% (204)	11.8% (126)	1.7% (18)	9.3% (99)
p__Cyanobacteria	0.3% (3)	0	0.2% (2)	0.4% (4)	0	0.1% (1)	0.4% (4)	0	0.1% (1)	0.1% (1)	0	0.4% (4)	0.3% (3)	0	0.1% (1)	0	0	0.2% (2)	0.2% (2)	0	0.2% (2)
p__Firmicutes	27.9% (313)	9.1% (102)	20.4% (229)	28.4% (316)	10.4% (115)	18.0% (200)	26.0% (259)	5.5% (55)	24.5% (244)	7.0% (77)	11.7% (128)	38.4% (421)	28.3% (302)	4.7% (50)	24.6% (263)	0.1% (1)	0.2% (2)	55.2% (507)	28.4% (303)	3.8% (41)	24.1% (257)
p__Fusobacteria	0.4% (4)	0	0.3% (3)	0.4% (4)	0	0.3% (3)	0.2% (2)	0	0.3% (3)	0	0	0.6% (6)	0.2% (2)	0	0.4% (4)	0	0	0.4% (4)	0.2% (2)	0	0.3% (3)
p__Proteobacteria	6.6% (74)	2.9% (32)	6.1% (68)	7.4% (82)	3.3% (37)	5.0% (55)	7.3% (73)	2.1% (21)	7.0% (70)	1.6% (18)	3.7% (40)	10.2% (111)	7.5% (80)	1.6% (17)	6.7% (97)	0	0.1% (1)	17.2% (158)	7.1% (76)	0.9% (10)	8.1% (86)
p__TM7	0	0	0.1% (1)	0	0.1% (1)	0	0	0	0.1% (1)	0	0.1% (1)	0	0	0	0.1% (1)	0	0	0.1% (1)	0	0	0.1% (1)
p__Tenericutes	0	0.1% (1)	0.1% (1)	0	0.1% (1)	0	0	0	0	0	0.1% (1)	0	0	0	0	0	0	0.1% (1)	0	0	0.1% (1)
p__Verrucomicrobi	0.1% (1)	0.2% (2)	0.4% (4)	0.2% (2)	0.2% (2)	0.3% (3)	0.2% (2)	0.2% (2)	0.3% (4)	0	0.3% (3)	0.4% (4)	0.2% (2)	0.1% (1)	0.4% (4)	0	0.1% (1)	0.7% (6)	0.1% (1)	0.1% (1)	0.5% (5)
